# Resveratrol: Anti-Obesity Mechanisms of Action

**DOI:** 10.3390/molecules191118632

**Published:** 2014-11-14

**Authors:** Leixuri Aguirre, Alfredo Fernández-Quintela, Noemí Arias, Maria P. Portillo

**Affiliations:** 1 Nutrition and Obesity Group, Department of Nutrition and Food Science, Faculty of Pharmacy, University of Basque Country (UPV/EHU) and Lucio Lascaray Research Center, 01006 Vitoria, Spain; E-Mails: leixuri.aguirre@ehu.es (L.A.); alfredo.fernandez@ehu.es (A.F.-Q.); noemi.arias@ehu.es (N.A.); 2 CIBERobn Physiopathology of Obesity and Nutrition, Institute of Health Carlos III (ISCIII), 01006 Vitoria, Spain

**Keywords:** resveratrol, adipogenesis, apoptosis, lipogenesis, lipolysis, thermogenesis, fatty acid oxidation

## Abstract

Resveratrol is a non-flavonoid polyphenol which belongs to the stilbenes group and is produced naturally in several plants in response to injury or fungal attack. Resveratrol has been recently reported as preventing obesity. The present review aims to compile the evidence concerning the potential mechanisms of action which underlie the anti-obesity effects of resveratrol, obtained either in cultured cells lines and animal models. Published studies demonstrate that resveratrol has an anti-adipogenic effect. A good consensus concerning the involvement of a down-regulation of C/EBPα and PPARγ in this effect has been reached. Also, *in vitro* studies have demonstrated that resveratrol can increase apoptosis in mature adipocytes. Furthermore, different metabolic pathways involved in triacylglycerol metabolism in white adipose tissue have been shown to be targets for resveratrol. Both the inhibition of *de novo* lipogenesis and adipose tissue fatty acid uptake mediated by lipoprotein lipase play a role in explaining the reduction in body fat which resveratrol induces. As far as lipolysis is concerned, although this compound *per se* seems to be unable to induce lipolysis, it increases lipid mobilization stimulated by β-adrenergic agents. The increase in brown adipose tissue thermogenesis, and consequently the associated energy dissipation, can contribute to explaining the body-fat lowering effect of resveratrol. In addition to its effects on adipose tissue, resveratrol can also acts on other organs and tissues. Thus, it increases mitochondriogenesis and consequently fatty acid oxidation in skeletal muscle and liver. This effect can also contribute to the body-fat lowering effect of this molecule.

## 1. Introduction

Resveratrol (*trans*-3,4',5-trihydroxystilbene) is a non-flavonoid polyphenol, belonging to the stilbenes group and produced naturally in several plants in response to injury or fungal attack [1]. It was first detected in the roots of white hellebore (*Veratrum grandiflorum*) and is found in the diet in the form of various foodstuffs, such as grapes, berries, red wine and nuts [2]. Although the molecule exists in two isoforms, *trans*-resveratrol and *cis*-resveratrol, the *trans* form, which is the preferred steric form in Nature, is relatively stable [3].

Results from pharmacokinetic studies carried out on animal and humans show that resveratrol has a low bioavailability. It is rapidly metabolized in intestine and liver by phase II enzymes [4]. The end products of this metabolism are mainly glucuronide and sulfate derivatives. Colon microflora can also produce the metabolite dihydroresveratrol [5].

Resveratrol is well known for its health benefits. It can prevent or reduce a wide range of diseases such as cardiovascular diseases, cancer and ischemic damage. It also decreases inflammation and oxidative stress, and prolongs the lifespan of various organisms [6,7]. More recently, resveratrol has been reported to improve glycaemic control in animals and subjects showing insulin resistance or diabetes [8,9,10] and to prevent obesity [8,11].

Although the mechanisms of action which explain this effect are not completely understood as yet, several metabolic pathways such as adipogenesis, apoptosis, lipogenesis, lipolysis, thermogenesis and fatty acid oxidation have been described in the literature as being effective targets for this polyphenol. The present review aims to compile evidence concerning the potential mechanisms of action underlying the anti-obesity effects of resveratrol obtained in cultured cells lines and animal models.

## 2. Effects of Resveratrol in Adipogenesis

Although some time ago it was believed that the number of adipocytes was constant over a lifetime, nowadays it is well known that adipocytes can be both gained and lost. Obesity can be due to an increase in number of adipocytes in adipose tissue (hyperplasia) to an increase of adipocyte size (hypertrophy) or both [12].

Adipocyte life cycles start with the differentiation of adipocytes from stem cells [13]. The first phase of this cycle is the growth phase, which is followed by growth arrest, clonal expansion, changes in gene expression leading to lipid storage, and finally cell death [14]. In this differentiation process CCAAT-enhancer-binding protein (C/EBPα), sterol regulatory element binding protein 1c (SREBP-1c) and peroxisome proliferator-activated receptor gamma (PPARγ) are required to induce changes from a fibroblastic cell shape to a spherical one. Of these, PPARγ is considered to be the main adipogenesis-inducing regulator.

C/EBPβ is an early regulatory factor which activates the expression of PPARγ and C/EBPα. These are in turn responsible for the activation of adipogenic genes throughout the process [15]. During the last phase of differentiation the adipocytes show a great increase in *de novo* lipogenesis, via increased expression and activity of fatty acid synthase (FAS), glucose-6-phosphate dehydrogenase (G6PDH) and malic enzyme (ME). This process is controlled by SREBP-1c. In this step, insulin sensitivity is enhanced due to an increase in insulin receptors (IR) and insulin-dependent glucose transporters (GLUT) [16].

Several studies in the literature have addressed the potential anti-adipogenic effect of resveratrol under *in vitro* conditions (Table 1). The vast majority of these studies have been carried out in 3T3-L1 pre-adipocytes, but other types of adipocytes have also been used.

**Table 1:** Effects of resveratrol on adipogenesis in *in vitro* studies.

Authors	Experimental Design	Effects
Kwon *et al.* (2012) [16]	3T3-L1 pre-adipocytes	↓ C/EBPα, PPARγ protein expression ↓ Mitotic clonal expansion
	25, 50 µM	
	24 h	
Chen *et al.* (2011) [17]	3T3-L1 pre-adipocytes	↓ PPARγ, C/EBPα, SREBP-1c mRNA expression
	20, 40, 80 µM	
	24, 48 h	
Rayalam *et al.* (2008) [18]	3T3-L1 pre-adipocytes	↓ PPARγ, C/EBPα, SREBP-1c and FAS, LPL mRNA
	25 µM6 days	
	6 days	
Kang *et al.* (2012) [19]	3T3-L1 pre-adipocytes	↓ Lipid accumulation ↓ C/EBPβ, PPARγ, C/EBPα, FABP4 protein expression
	10, 20, 40 µM	
	2, 4, 6 days	
Lasa *et al.* (2012) [20]	3T3-L1 pre-adipocytes	↓ Triacylglycerol content by resveratrol, *trans*-resveratrol-4-*O*'-glucuronide, *trans*-resveratrol-3-*O*-sulfate ↓ C/EBPβ mRNA ↓ C/EBPα, PPARγ mRNA by *trans*-resveratrol-3-*O*-sulfate
	1, 10, 25 µM resveratrol and its metabolites (*trans*-resveratrol-3-*O*-glucuronide, *trans*-resveratrol-4-*O'*-glucuronide, *trans*-resveratrol-3-*O*-sulfate)	
	8 days	
Hu *et al.* (2014) [21]	3T3-L1 pre-adipocytes	↑ C/EBPα, PPARγ and FABP4 mRNA expression at 1 and 10 µM ↓ C/EBPα, PPARγ and FABP4 mRNA expression at 50 and 100 µM
	1, 10, 50, 100 µM	
	7 days	
Carpéné *et al.* (2014) [22]	3T3 F442A pre-adipocytes	↓ Mitotic clonal expansion
	20 µM	
	8 days	
Bai *et al.* (2008) [23]	Pig primary pre-adipocytes	↑ SIRT-1 mRNA ↓ FOXO1 and PPARγ2 mRNA expression
	100 µM	
	72 h	
Fisher-Posovszky *et al.* (2010) [24]	Human Simpson-Golabi-Behmel Syndrome pre-adipocytes	↓ PPARγ, GLUT4 and FAS mRNA expression
	10, 30, 50, 100 µM	
	4 days	

C/EBP: CCAAT/enhancer-binding protein; PPARγ: Peroxisome proliferator-activated receptor gamma; SREBP1: Sterol regulatory element binding protein 1; FAS: Fatty acid synthase; LPL: Lipoprotein lipase; FABP4: Fatty acid binding protein.

In a study carried out in 3T3-L1 pre-adipocytes by Chen *et al.*, a wide range of resveratrol concentrations (10, 20, 40 and 80 µM) was used for cell incubation for 48 h [17]. The authors observed that, with the exception of the lowest concentration (10 µM), the other concentrations reduced lipid accumulation is these cells in a dose-dependent manner. Moreover, the authors analyzed gene and protein expression of PPARγ, C/EBPα and SREBP-1c, three transcriptional factors involved in adipogenesis regulation. All the concentrations, except 10 µM, reduced both gene and protein expression of these transcriptional factors. In view of these results, the authors concluded that resveratrol reduced adipogenesis. In order to gain insight into the mechanism of action underlying the anti-adipogenic effect of resveratrol, as an indicator of enzyme activation the phosphorylation of AMPK was measured. The results showed that resveratrol activated AMPK. Nevertheless, due to the fact that no significant correlation was found between the level of AMPK phosphorylation and the expression of adipogenic transcriptional factors, the authors suggested that further research was needed to clarify the role of AMPK in the anti-adipogenic effect of resveratrol.

Kwon *et al.* also treated 3T3-L1 pre-adipocytes, but in this case for 24 h [16]. These authors incubated the cells with resveratrol at concentrations of 25 and 50 µM, and reported that the presence of this polyphenol in culture medium inhibited adipogenesis. Consistent with this result, a down-regulation of C/EBPα and PPARγ protein expression was observed. These results are in good accordance with those reported by Chen *et al.* [17], and show that 1 day of incubation is enough for resveratrol to show its anti-adipogenic effect.

It has been proposed that resveratrol drives several of its beneficial effects by targeting and activating the NAD+-dependent protein deacetylase SIRT-1, although the reported results have been somewhat controversial [25,26,27,28]. In the study by Kwon *et al.*, the authors suggested that the anti-adipogenic effect of resveratrol was unlikely to be dependent on SIRT-1 because sirtinol, an inhibitor of this deacetylase, had no effect on reversing the inhibition of adipogenesis induced by the polyphenol [16].

The effects of resveratrol on cell differentiation have also been assessed using incubation periods of up to 8 days [18,19,20,21,22,24]. Thus, Rayalam *et al.* [18] described that resveratrol at concentrations of 25 and 50 µM significantly decreased lipid accumulation in 3T3-L1 pre-adipocytes after 6 days of incubation. A dose-response pattern was observed in this range of concentrations. When they analyzed cell viability in these cells, they observed that both doses of resveratrol decreased this parameter. In addition, the authors measured gene expression of PPARγ, C/EBPα and SREBP-1c, and reported that the three genes were down-regulated when pre-adipocytes were incubated with 25 µM of resveratrol for 6 days. Based on these results, which are in line with those reported by Kwon *et al.* [16] and Chen *et al.* [17], the authors suggested that the reduction in lipid accumulation induced by resveratrol was due to both decreased cell viability and reduced adipogenesis [18].

Moreover, Kang *et al.* [19] proposed different incubation periods (2, 4 and 6 days) with resveratrol at 10, 20, and 40 µM. Lipid accumulation was inhibited by 6.2%, 7.6%, and 8.5% after 2 days of incubation with resveratrol at 10, 20, and 40 µM, respectively. When the treatment period was extended to 4 days, pre-adipocyte differentiation was reduced by 9.4% with 10 µM of resveratrol and by 38.6% with 40 µM resveratrol. Finally, the addition of resveratrol for 6 days did not cause further inhibition in pre-adipocyte differentiation, at the dose of 10 µM. In the case of the highest dose (40 µM) a slight increase in adipogenesis inhibition was observed (40.3%). Protein expression of C/EBPβ, C/EBPα, PPARγ and FABP4 was measured and a dose-dependent reduction was observed. The authors concluded that resveratrol inhibited adipocyte differentiation and proliferation by modulating the protein expression of these transcription factors. In this study the dose of 10 µM of resveratrol was effective in reducing adipogenesis. However, Chen *et al.* [17] did not find this effect at the same concentration, even though the culture conditions were similar in both studies. The reason for this discrepancy is not clear.

In a study performed in our group [20] we treated 3T3-L1 pre-adipocytes from day 0 to day 8 of differentiation with 10 and 25 µM of resveratrol, concentrations previously used by other authors, but also with a lower dose (1 µM) which is closer to the concentrations of this polyphenol found in plasma and tissues. The doses of 1 and 10 µM had no effect on triacylglycerol content. By contrast, a reduction in this parameter was observed after the addition of 25 µM of resveratrol. This effect was accompanied by a decrease in mRNA levels of C/EBPβ, without changes in C/EBPα and PPARγ gene expressions.

As indicated in the Introduction section, resveratrol bioavailability is very low and only a small proportion reaches plasma and tissues. The concentrations of glucuronide and sulfate metabolites are relatively higher than those of the parent compound [29]. Interestingly, despite the low concentrations of resveratrol found in plasma and tissues, beneficial effects induced by this molecule have been reported in the literature [30]. This may suggest that some resveratrol metabolites could in fact show biological activities. In this context, in the above mentioned study from our group [20] the effects of the main metabolites of resveratrol (*trans*-resveratrol-3-*O*-glucuronide, *trans*-resveratrol-4-*O'*-glucuronide, and *trans*-resveratrol-3-*O*-sulfate) were assessed. As in the case of resveratrol, the doses of 1 and 10 µM had no effect. By contrast, a reduction in triacylglycerol content was observed after the addition of 25 µM of *trans*-resveratrol-4-*O*-glucuronide and *trans*-resveratrol-3-*O*-sulfate. With regard to *trans-*resveratrol-3-*O*-glucuronide, a reduction was also observed, but this did not reach statistical significance. The three metabolites reduced mRNA levels of C/EBPβ. As far as C/EBPα and PPARγ gene expressions were concerned only *trans*-resveratrol-3-*O*-sulfate induced significant reductions.

Despite this general consensus concerning the inhibition of adipogenesis induced by resveratrol, Hu *et al.*, have recently reported increased adipogenesis induced by resveratrol under specific experimental conditions [21]. In this study the authors analyzed the effects of 1, 10, 50 and 100 µM of this polyphenol, added to the medium for 7 days, by using a modified differentiation cocktail containing cortisone, a weaker glucocorticoid receptor agonist than dexamethasone. Resveratrol at 1 and 10 µM enhanced 3T3-L1 adipocyte differentiation in a dose dependent manner. By contrast, at 50 and 100 µM it suppressed adipocyte differentiation. The effect of resveratrol on adipocyte differentiation was confirmed by mRNA expression of differentiation markers, PPARγ, C/EBPα and FABP4. The authors proposed that the adipogenic effects may be mediated by the activation of glucocorticoid receptors.

By comparing the reported effects of resveratrol at 1 µM, it can be observed that whereas Hu *et al.* [21] observed an adipogenic effect, in our study [20] we did not find any changes when using quite similar incubation periods. As far as the dose of 10 µM is concerned, several studies found it ineffective [17,20], Kang *et al.* [19] reported an anti-adipogenic effect, and Hu *et al.* [21] showed an adipogenic action. The results obtained by using concentrations greater than 10 µM show that resveratrol acts as an anti-adipogenic molecule in all the reported studies. Taking into account the results published by Hu *et al.* [21] it seems that the composition of the incubation medium is an important factor when the effects of resveratrol on adipogenesis are considered, at least in the range of 1 to 10 µM.

Carpéné *et al.* [22] used a type of murine pre-adipocyte (3T3 F442A) which depends only on insulin, but not on corticoids, to differentiate into adipocytes. They observed that when cells were cultured for 8 days with 20 µM resveratrol, clonal expansion and lipid accumulation were significantly reduced. These results show that the effect of resveratrol on this specific type of 3T3 pre-adipocytes is similar to that induced in the commonly used 3T3-L1 cells.

Other studies have been performed using primary adipocyte cultures. Bai *et al.* incubated pig primary pre-adipocytes with 100 µM of resveratrol for 72 h [23]. Resveratrol increased gene expression of SIRT-1, while FoxO1 and PPARγ2 mRNA levels notably decreased. The authors concluded that the activation of SIRT-1 could inhibit the proliferation and differentiation of pig pre-adipocytes. They proposed that SIRT-1 may directly control the expression of adipocyte gene markers, or indirectly through its effect on FoxO1. Due to the fact that only a high dose of resveratrol was used in this study, it is not possible to know whether pig pre-adipocytes were more or less sensitive to the action of this polyphenol than murine 3T3-L1 pre-adipocytes.

Finally, Fisher-Posovszky *et al.* used human Simpson-Golabi-Behmel Syndrome (SGBS) pre-adipocytes for their study [24]. They incubated these cells with 10, 30, 50 and 100 µM of resveratrol for the first four days of adipogenic differentiation. Concentrations higher than 10 µM induced the inhibition of this process; the maximal inhibitory effect was observed at 100 µM. On day 10 of differentiation, a significant reduction in the expression of the adipogenic transcription factor PPARγ, and several marker genes, such as GLUT4 and FAS, was observed.

**Figure 1:**
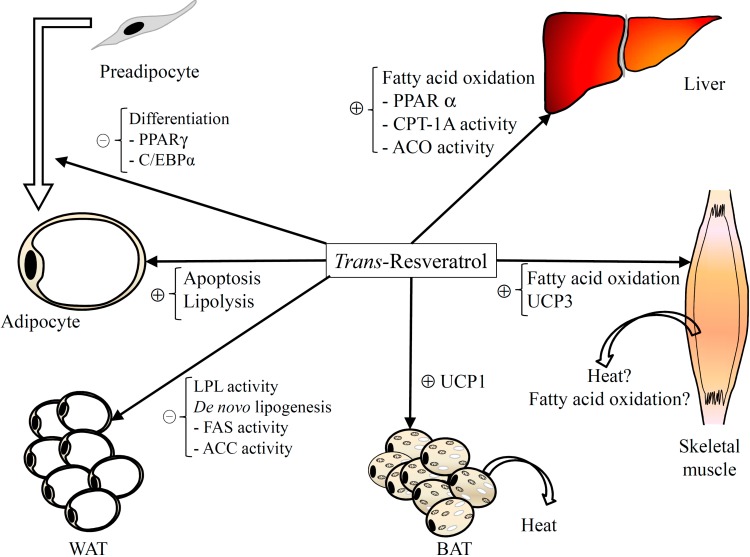
Major mechanisms involved in the anti-obesogenic effect of resveratrol. ACC: Acetyl-CoA carboxylase; ACO: Acyl-CoA oxidase; BAT: Brown adipose tissue; C/EBP: CCAAT/enhancer-binding protein; CPT: Carnitine palmitoyltransferase; FASN: Fatty acid synthase; LPL: Lipoprotein lipase; PPAR: Peroxisome proliferator-activated receptor; UCP: Uncoupling protein; WAT: White adipose tissue.

In summary, all the published studies demonstrate that resveratrol shows anti-adipogenic effects at concentrations in the range of 20–100 μM (Figure 1). This effect is observed in murine, porcine and human pre-adipocytes. There are marked differences among the reported studies in the treatment period length (24 h to 8 days), but in all cases resveratrol is able to inhibit adipogenesis. There is a good consensus concerning the mechanism of action underlying this effect. Thus, resveratrol inhibits C/EBPβ, the early regulatory factor of adipogenesis. Moreover, the expression of C/EBPα and PPARγ, required to induce changes in the cell shape from a fibroblastic to a spherical shape, is also reduced by this polyphenol. These changes can be mediated by SIRT-1.

## 3. Effects of Resveratrol in Apoptosis

Apoptosis is a process of programmed cell suicide, inherent to most nucleated cells, which is activated when they are deprived of essential pro-survival factors [31,32]. Moreover, apoptosis can be induced by certain types of severe cell distress or by specific death-inducing ligands [32]. This process is mediated by caspases [31], which can be classified as initiator caspases (caspase 8 and 9) and executioner or effector caspases (caspase 3 and 7) [33].

In mammals, caspases can be activated by two cellular signaling pathways: the intrinsic pathway, which controls the release of specific caspase-activating factors from mitochondria [34] and the extrinsic pathway, which transmits signals from extracellular death ligands across the plasma membrane to engage the intracellular caspase machinery. These two pathways often act directly and/or indirectly to reinforce one another [32].

Several studies have addressed the potential apoptotic effect of resveratrol (Table 2). In these studies 3T3-L1 mature adipocytes incubated with 50 and 100 µM of resveratrol for 24 and 48 h have been used [18,35,36,37]. These studies showed that exposure of these cells to 50 µM of resveratrol for 24 or 48 h did not increase apoptosis. However, this phenomenon was observed at a dose of 100 µM for 24 [36] or 48 h [35].

With regard to pre-adipocytes, Chen *et al.* and Pang *et al.* by using 3T3-L1 and porcine pre-adipocytes respectively, reported an apoptotic effect of resveratrol at concentrations in the range of 40–50 µM for 48 h [38,39].

Some authors proposed that apoptosis in 3T3-L1 mature adipocytes was mediated via SIRT-1/AMPK axis [38]. However, Pang *et al.* [39], reported that apoptosis is SIRT-1 independent. This discrepancy could be due to the cell lines, since in this study the authors used porcine pre-adipocytes while Chen *et al.* used 3T3-L1 adipocytes. Moreover, Pang *et al.* proposed that resveratrol treatment increases caspase-3 protein, a critical executor during apoptosis.

**Table 2:** Effects of resveratrol on apoptosis.

Authors	Experimental Design	Effects
Yang *et al.* (2008) [35]	3T3-L1 mature adipocytes	↓ Cell viability ↑ Apoptosis
	50, 100 µM	
	24, 48 h	
Chen *et al.* (2012) [38]	3T3-L1 pre-adipocytes	↑ Apoptosis
	20, 40, 80 µM	
	24, 48 h	
Pang *et al.* (2013) [39]	Porcine pre-adipocytes	↑ Apoptosis
	50, 100, 200 and 400 µM	
	48 h	

Consequently, these results show an apoptotic effect of resveratrol in adipocytes (Figure 1), but it is important to indicate that this effect is observed at quite high concentrations of this polyphenol. Maturing adipocytes seem to be more sensitive to resveratrol, since the concentrations needed to promote the apoptotic response are lower than those in mature adipocytes.

## 4. Effects of Resveratrol on *De Novo* Lipogenesis and Lipoprotein Lipase Function in Adipose Tissue

Two important metabolic pathways involved in the control of fat accumulation in adipose tissue are *de novo* lipogenesis and fatty acid uptake from circulating triacylglycerols. *De novo* lipogenesis is the synthesis of esterified fatty acids, from acetyl-CoA, used as substrates in the synthesis of triacylglycerols. Two rate-limiting enzymes in this process are acetyl-CoA carboxylase (ACC) and fatty acid synthase (FASN). The former catalyzes the irreversible carboxylation of acetyl-CoA to produce malonyl-CoA and the latter, which is in fact an enzymatic complex, catalyzes the synthesis of palmitate from acetyl-CoA and malonyl-CoA into long-chain saturated fatty acids. Moreover, malic enzyme (ME) and glucose-6 phosphate dehydrogenase (G6PDH) provide NADPH to fatty acid synthesis reactions. SREBP-1c is one of the main transcription factors that regulate the expression of ACC and FASN [40].

In adipose tissue, fatty acids for triacylglycerol synthesis can also come from circulating triacylglycerol-rich lipoproteins (chylomicrons and very low-density lipoproteins). In this tissue the enzyme lipoprotein lipase (LPL), which is attached to the luminal surface of endothelial cells in capillaries, hydrolyzes lipoprotein triacylglycerols into two free fatty acids and one monoacylglycerol molecule [41]. The transcriptional factor which controls the expression of LPL is PPARγ [42].

In the next paragraphs, the effects of resveratrol on these two metabolic pathways reported in the literature in both *in vitro* and *in vivo* experiments are described (Table 3). In a study carried out by our group we treated mature 3T3-L1 adipocytes with 1, 10 or 25 µM of resveratrol for 24 h. Exposure of these cells to 1 and 10 µM resveratrol triggered a significant reduction in intracellular triacylglycerol content (52.7% and 60.0% respectively). The highest dose (25 µM) did not produce any additional delipidating effect. In this study, gene expression of ACC and FASN was studied in adipocytes treated with 10 µM of resveratrol, and no changes were observed. This suggests that lipogenic pathway was not affected in these cells [21].

**Table 3:** Effects of resveratrol on *de novo* lipogenesis and lipoprotein lipase in *in vitro* and *in vivo* studies.

Authors	Experimental Design	Effects
Lasa *et al.* (2012) [22]	3T3-L1 pre-adipocytes	↓Triacylglycerol content ↓ACC mRNA expression ↑SIRT-1 mRNA expression
	1, 10, 25 µM resveratrol and its metabolites (*trans*-resveratrol-3-*O*-glucuronide, *trans*-resveratrol-4-*O'*-glucuronide, *trans*-resveratrol-3-*O*-sulfate)	
	24 h	
Szkudelska *et al.* (2009) [43]	Rat primary adipose culture from epididymal	↓ Basal lipogenesis from glucose
	62.5, 125, 250 µM	
	90 min	
Rivera *et al.* (2009) [44]	Male Zucker rats	↓Abdominal fat ↑pACC protein expression ↑AMPK
	10 mg/kg body weight/day	
	8 weeks	
Gómez-Zorita *et al.* (2013) [45]	Male Zucker rats	↓ Final body weight and internal fat depots ↓ G6PDH = FAS, ME ACC ↓ LPL activity, = LPL mRNA expression
	15 mg/kg body weight/day	
	6 weeks	
Nagao *et al.* (2013) [46]	OLETF rats	↓ Abdominal fat depot = FAS mRNA expression
	0.5% resveratrol	
	4 weeks	
Alberdi *et al.* (2011) [47]	Male Sprague-Dawley rats	↓ Perirenal, epididymal, mesenteric and subcutaneous fat depots ↓ ACC, FAS, G6PDH activities
	30 mg/kg body weight/day	
	6 weeks	
Arias *et al.* (2014) [48]	Male Wistar rats	↓ FAS and LPL activities
	30 mg/kg body weight/day	
	6 weeks	
Kim *et al.* (2011) [49]	C57BL/6J mice	↓ Adipose tissue weight ↓ SREBP-1c, FAS mRNA expression = PPARγ mRNA expression
	0.4% resveratrol	
	10 weeks	
Cho *et al.* (2012) [50]	C57BL/6J mice	↓ Visceral adipose tissue weight ↓ FAS, G6PDH
	0.005 and 0.02% resveratrol	
	10 weeks	
Qiao *et al.* (2014) [51]	Kumming mice	↓ ACC, FAS mRNA expression
	200 mg/kg/d	
	12 weeks	
Azorin-Ortuño *et al.* (2012) [52]	PBMCs of pigs	↓ LPL mRNA expression
	0.11 mg/kg body weight/d	
	12 months	

ACC: acetyl-CoA carboxylase; SIRT1: sirtuin 1; pACC: phosphorylated ACC; G6PDH: glucose-6 phosphate dehydrogenase; FAS: fatty acid synthase; ME: malic enzyme; LPL: lipoprotein lipase; SREBP1: sterol regulatory element binding protein 1.

In this study the effects of the three main resveratrol metabolites were also assessed. Mature 3T3-L1 adipocytes treated with 1 µM of *trans*-resveratrol-3-*O*-glucuronide showed a tendency towards lower values, and the other two metabolites did not show any delipidating effect. At 10 µM, the glucuronide metabolites showed delipidating effect. By contrast, *trans*-resveratrol-3-*O*-sulfate did not induce significant changes. At the highest concentration (25 µM) additional delipidating effects were not observed. With regard to the expression of lipogenic enzymes, which was measured in adipocytes treated with 10 µM of resveratrol metabolites, only FAS was reduced by *trans*-resveratrol-3-*O*-glucuronide.

Szkudelska *et al.* carried out an interesting study in which incubating adipocytes, isolated from rat epididymal adipose tissue, with resveratrol revealed its inhibitory influence on glucose conversion to lipids. They observed that 125 and 250 µM of resveratrol significantly decreased basal lipogenesis from glucose, whereas 62.5 µM was ineffective. Similar effects were observed when lipogenesis was stimulated by insulin [43].

Although several data have been obtained in *in vitro* studies, the vast majority of the studies devoted to analyzing the effects of resveratrol on *de novo* lipogenesis and LPL have been conducted in *in vivo* experiments. Rivera *et al.* [44] analyzed the effects of resveratrol on body fat accumulation in 13-week-old Zucker rats. They compared the effects of this polyphenol on obese rats (*fa/fa*), which show metabolic alterations similar to those found in human metabolic syndrome, and on their lean littermates (*Fa/Fa*). Obese and lean animals were divided in two groups, one control group and a group treated with resveratrol at a dose of 10 mg/kg body weight/d for 8 weeks. Resveratrol was orally administered by gavage. Polyphenol administration did not induce changes in food intake or body weight in obese or lean rats. Obese rats treated with resveratrol, but not lean rats, showed a significantly lower content of abdominal fat. As far as enzymes related to *de novo* lipogenesis are concerned, this polyphenol significantly increased protein expression of phosphorylated ACC. Taking into account that the phosphorylated isoform of ACC is the inactive one, these results show that resveratrol decreased ACC activity and thus reduced *de novo* lipogenesis [44].

It has been reported that resveratrol leads to the activation of AMP-activated protein kinase (AMPK) [28,53,54,55], a major metabolic-sensing protein which has been implicated in the prevention of metabolic disorders. In this study the inactivation of ACC was mediated by AMPK, which was activated by phosphorylation in resveratrol-treated rats. In good accordance with the effects of this treatment on body fat, changes in ACC activity were only observed in obese rats.

In our group we also performed an experiment in obese (*fa/fa*) Zucker rats. In this study, rats were treated with resveratrol at a dose of 15 mg/kg body weight/d for 6 weeks. The polyphenol was orally administered by gavage [45]. Resveratrol treatment significantly reduced final body weight and internal adipose tissues, without significant changes in food intake. G6PDH activity was significantly reduced in epididymal adipose tissue taken from resveratrol-treated rats, but no significant changes were observed in FAS and ME activities. Similarly, the ratio of phosphorylated ACC to total ACC was not modified in the treated group, and thus it can be proposed that enzyme activity was not altered. These results are not in good accordance with those previously reported by Rivera *et al.* [44]. The reasons that can justify this discrepancy are not clear. The main difference between both experimental designs is the experimental period length. Perhaps the longer treatment enabled Rivera *et al.* to find a reduction in ACC activity.

For their study, Nagao *et al.* [46] used Otsuka Long-Evans Tokushima Fatty (OLETF) rats, another strain of rat which develops a syndrome with multiple metabolic and hormonal disorders. The authors observed that when animals were fed a standard diet supplemented with 0.5% resveratrol for 4 weeks, accumulation of abdominal white adipose tissue was noticeably prevented. In order to gain more insight into this effect the authors analyzed the mRNA expression of FAS, among other genes, and they did not observe changes. The activity of lipogenic enzymes was not measured in this study [46].

Other studies, also performed in rats, have used dietetic models of obesity instead of genetic models. In a study from our group, conducted using Sprague-Dawley rats fed an obesogenic diet (high-fat, high-sucrose diet), we observed that after 6 weeks of treatment with resveratrol at a dose of 30 mg/kg body weight/d, rats showed a significant reduction in adipose tissue size from different anatomical locations (perirenal, epididymal, mesenteric and subcutaneous), without changes in food intake and body weight. As far as *de novo* lipogenesis was concerned, we measured the activity of ACC, FAS, ME and G6PDH, as well as the expression of ACC, FAS and SREBP-1c, the transcription factor which regulates these two enzymes [47]. Resveratrol-treated rats showed significantly reduced activities of ACC, FAS and G6PDH. However, no significant changes were induced by resveratrol feeding in the expression of lipogenic enzymes (ACC and FAS) or SREBP-1c. These results suggest that, under these experimental conditions, *de novo* lipogenesis contributed to the reduction in body fat induced by resveratrol. Further, it can be pointed out that the effects on ACC and FAS occur at a post-transcriptional level. Using the same experimental design, but in this case another strain of rat (Wistar), we also observed significant reductions in the activities of FAS and LPL induced by resveratrol treatment [48].

The mouse has also been used as an experimental animal model in studies focusing on the anti-obesity effects of resveratrol. In a study by Kim *et al.* [49] C57BL/6J mice were fed a high-fat diet, either with or without resveratrol supplementation (0.4%), for 10 weeks. Mice which received the supplemented diet showed significantly reduced adipose tissue weights. Moreover, a significant reduction in gene expression of SREBP-1c and its target gene FAS was observed in adipose tissue, suggesting that a reduction in *de novo* lipogenesis was involved in the body-fat lowering effect of resveratrol [49].

A later experiment by Cho *et al.* [50] used the same mouse strain but two far lower levels of resveratrol supplementation in the diet (0.005% and 0.02%), than those used in the experiment reported by Kim *et al.* [49]. After 10 weeks of treatment total visceral weight was significantly reduced, although the low dose appeared to be more effective than the higher dose. Resveratrol significantly reduced FAS activity in adipose tissue. Furthermore, mice supplemented with the low dose of this molecule showed a significant decrease in G6PDH activity when compared with mice fed the high-fat diet alone [50]

In another strain of mice, Qiao *et al.* [51] found results in line with those reported by Kim *et al.* [49], but in this case using a very high dose of resveratrol. They treated Kumming mice with resveratrol at a dose of 200 mg/kg body weight/d for 12 weeks, and they observed that this polyphenol prevented the increase induced by high-fat feeding in gene of expression of ACC and FAS [51].

As far as the expression and activity of LPL is concerned, very few data have been reported in the literature. In our study carried out in obese Zucker rats, rats treated with resveratrol (15 mg/kg body weight/d) showed a significant reduction in LPL activity, but not in LPL gene expression [56]. This means that the change induced by resveratrol in this enzyme, which was involved in the body fat-lowering effect of this polyphenol, was post-transcriptional. Similar results were obtained in another study reported by our group, already mentioned in this review, conducted in Sprague–Dawley rats fed an obesogenic diet. In this case gene expression of PPARγ, the transcriptional factor which controls LPL expression, was also measured and significant changes were not observed [47].

Finally, Azorin Ortuño *et al.* [52] used a microarray technique to analyze the expression of several genes involved in obesity in PBMCs (peripheral blood mononuclear cells) from pigs treated with resveratrol. Peripheral blood mononuclear cells express approximately 80% of the genes expressed in other tissues that can be altered in response to internal and external signals, and can be used to investigate biological changes and novel biomarkers. Thus, PBMC gene expression has been used to characterize metabolic changes caused by the diet [57]. In this experiment the authors observed a significant reduction in LPL expression in animals treated with resveratrol at a dose of 0.11 mg/kg body weight/d for 12 months [52].

Taken together these results suggest that *de novo* lipogenesis, as well as adipose tissue fatty acid uptake mediated by LPL, are targets for resveratrol. The inhibition of these two metabolic processes goes towards explaining to justify the reduction in body fat induced by this polyphenol (Figure 1). As far as *de novo* lipogenesis is concerned, reduced FAS expression and activity and/or decreased ACC activation have been reported. With regard to LPL, reductions in both gene expression and activity have been observed. As a consequence, it can be stated that resveratrol can regulate these metabolic pathways at transcriptional and post-transcriptional levels. The discrepancies found in the literature can be attributed to differences in various aspects of the experimental designs of the reported studies, namely experimental period length, resveratrol dose, animal species, and animal strain.

## 5. Effects of Resveratrol on Lipolysis

Lipolysis implies the breakdown of triacylglycerols in adipocytes and the release of glycerol and fatty acids. Two major enzymes promote the catabolism of stored triacylglycerols: adipose triglyceride lipase (ATGL) and hormone-sensitive lipase (HSL) [58,59]. ATGL selectively performs the first step in triacylglycerol hydrolysis yielding free fatty acids and diacylglycerols which, in turn, are substrates for HSL [59].

HSL activation depends on its protein kinase A (PKA)-dependent phosphorylation, which is mediated via the accumulation of cAMP [18]. Further, SIRT-1 has been reported to trigger lipolysis and to induce fat mobilization [60]. Moreover, it has been suggested that the sympathetic nervous system contributes to the stimulation of SIRT-1 activity [43,61].

The effects of resveratrol on lipolysis have been studied either by *in vitro*, *ex vivo* or by *in vivo* approaches (Table 4). As far as *in vitro* studies are concerned, Shan *et al.* [62] obtained porcine adipocytes from the subcutaneous adipose tissue of piglets (5 to 7 days of age). The mature adipocytes were incubated with either 25 or 50 μM resveratrol for 24 or 48 h. The authors observed increased SIRT-1 and ATGL gene expressions associated with an enhanced glycerol release to the medium. This effect occurred solely with the higher concentration of resveratrol, without a time-response pattern [62].

**Table 4:** Effects of resveratrol on lipolysis in *in vitro* and *in vivo* studies.

Authors	Experimental Design	Effects
Lasa *et al.* (2012) [22]	3T3-L1 adipocytes 1, 10, 25 µM resveratrol, resveratrol-4'-*O*-glucuronide, resveratrol-3-*O*-glucuronide and resveratrol-3-*O*-sulfate 24 h	↓ TG content: resveratrol, resveratrol-4'-*O*-glucuronide, resveratrol-3-*O*-glucuronide ↑ SIRT1 mRNA: resveratrol, resveratrol-4'-O-glucuronide, resveratrol-3-*O*-glucuronide ↑ PGC-1α mRNA: resveratrol ↑ ATGL mRNA: resveratrol ↑ HSL mRNA: resveratrol-4'-*O*-glucuronide
Szkudelska *et al.* (2009) [43]	Rat adipocytes 1, 10, 100 µM 90 min	↑ Epinephrine-stimulated glycerol release (100 µM) ↑ Anti-lipolytic effect of insulin (1, 10, 100 µM) ↑ Epinephrine-stimulated glycerol release in resveratrol pre-incubated adipocytes (100 µM)
Gómez-Zorita *et al.* (2013) [45]	Obese Zucker (*fa*/*fa*) rats	↑ HSL mRNA = ATGL and SIRT-1 mRNA
	15 mg/kg body weight/day (gavage)	
	Chow diet	
	6 weeks	
Alberdi *et al.* (2011) [47]	Male Sprague-Dawley rats	↑ HSL mRNA = ATGL mRNA
	High-fat, high sucrose diet	
	30 mg/kg body weight/day	
	6 weeks	
Shan *et al.* (2013) [62]	Porcine adipocytes	↑ Glycerol release at 50 μM ↑ SIRT1 and ATGL mRNA at 50 μM
	25 or 50 μM r	
	24, 48 h	
Lasa *et al.* (2012) [63]	3T3-L1 adipocytes 100 µM 12, 24, 48 h	↑ Free fatty acid release ↑Isoproterenol-stimulated glycerol release (100 µM) ↑↑ ATGL mRNA and protein expression = HSL mRNA levels
	SGBS adipocytes	↑ ATGL mRNA and protein expression = HSL mRNA levels
	100, 200 µM	
	12, 24, h	
	C57BL/6 mice (WT, ATGL KO, HSL KO)	↑ Free fatty acids release in WT and HSL KO mice = Free fatty acids release in ATGL KO mice
	Epididymal fat pad adipocytes	
	100 µM	
	8 h	
Rosenow *et al.* (2012) [64]	SGBS adipocytes	↓ Intracellular TG content ↑ Glycerol release at 75 μM
	5, 50, 75, 100, and 200 μM	
Pedersen *et al.* (2008) [65]	Human (obese women) abdominal subcutaneous adipose tissue fragments	↑ Epinephrine-stimulated glycerol release
	50 μM	
	2 h	
Gómez-Zorita *et al.* (2013) [66]	Human (overweight women) adipocytes	↑ Isoprenaline-stimulated glycerol release (10 μM)
	0.1, 10 μM	
	2 h	

TG: Triacylglycerol; SIRT-1: Sirtuin 1; PGC-1α: PPAR-γ co-activator-1 α; ATGL: Adipose triglyceride lipase; HSL: Hormone sensible lipase; WT: Wild type; KO: knock-out; SGBS: Human Simpson-Golabi-Behmel Syndrome.

Szkudelska *et al.* in a study carried out in adipocytes isolated from the epididymal adipose tissue of Wistar rats, observed that resveratrol on its own (100 μM) failed in promoting lipolysis, even when adipocytes were pre-incubated for 60 min with this phenolic compound. However, the polyphenol was shown to exert synergistic action with epinephrine (0.5 μM), enhancing its lipolytic capacity. These resveratrol-induced changes in the lipolytic response to epinephrine were not mediated by estrogen receptors, since the addition of an estrogen receptor blocker (ICI 182,780; 1 nM) to the medium did not alter the lipolytic response [43].

In a study from our laboratory, we observed lower triacylglycerol accumulation in 3T3-L1 adipocytes treated with resveratrol (100 μM) for 12, 24 or 48 h. Importantly, resveratrol treatment led to a significantly higher release of free fatty acids (FFA), as a measure of lipid mobilization, after the three treatment periods analyzed. Further, after incubation for 24 h, resveratrol not only induced higher FFA release in basal conditions in this cell line, but also when lipolysis was stimulated with isoproterenol (10 μM), a β-adrenergic agonist. When searching for the mechanisms underlying these effects, we observed that resveratrol definitively enhanced both ATGL mRNA levels and protein expression, with no statistically significant changes for HSL. In this report, we also studied the lipolytic effect of resveratrol (100 μM) on human SGBS adipocytes. Interestingly, resveratrol treatment had no effect on lipid mobilization in basal conditions in SGBS cells after 12 or 24 h of treatment. However, it induced higher FFA release when lipolysis was stimulated with 10 μM isoproterenol. In this cell line, resveratrol solely increased ATGL mRNA and protein expressions with no changes for HSL [63].

In the same study, AMP kinase and HSL activities were inhibited by using compound C and 76-0079 (HSLi) respectively. When compound C was added to the incubation medium in 3T3-L1 adipocytes, the increase produced in FFA release by isoproterenol was blocked. In addition, when HSL activity in SGBS cells was inhibited by HSLi, the increase induced by resveratrol in the lipolytic action of isoproterenol was maintained. These results show that FFA release induced by resveratrol is mediated by AMPK. In order to analyze whether changes in ATGL were induced at a transcriptional or a post-transcriptional level, gene and protein expressions of ATGL were measured and significant increases were observed in both cases [63].

Finally, we also performed an *ex vivo* experiment with epididymal adipose tissue removed from wild-type (WT) C57BL/6 mice and ATGL knockout (ATGL KO) or HSL knockout (HSL KO) mice [63]. The results obtained were in the same line as those provided by th*e in vitro* studies. Indeed, when adipose tissue from WT and HSL KO mice was incubated for 8 h with resveratrol, FFA release was significantly increased, suggesting enhanced lipolysis. Contrastingly, no changes were observed in tissues of ATGL KO mice.

As previously mentioned in this review, in another experiment carried out by our group we assessed the delipidating effects of resveratrol and some of its metabolites. In this study we analyzed the effects of this molecule on lipase expression. Whereas resveratrol significantly increased the expression of ATGL at a dose of 10 µM, the two glucuronide metabolites only showed a tendency towards increased values of this gene expression. Furthermore, only *trans*-resveratrol-4'-*O*-glucuronide increased HSL lipase expression. As far as SIRT-1 is concerned, only resveratrol and its glucuronide metabolites significantly up-regulated its gene expression [22].

Rosenow *et al.* incubated mature human SGBS adipocytes with different resveratrol concentrations (5, 50, 75, 100, and 200 μM). They observed that 75 μM was the lowest concentration that produced a significant triacylglycerol reduction accompanied by an increase in glycerol release. Resveratrol-treated SGBS adipocytes showed a significant decrease in intracellular triacylglycerol content. Based on the increase in basal glycerol release, the authors suggested that the decreased lipid content observed might be, at least in part, the result of increased intracellular lipolysis [64].

Pedersen *et al.* [65] studied the effect of resveratrol on lipolysis in abdominal subcutaneous adipose tissue fragments stimulated with either epinephrine (0.1 μM) alone or epinephrine and resveratrol (50 μM) together for 2h. Tissue fragments were obtained by liposuction from lean women before and after 6 days of total fasting. The authors found that epinephrine led to an increased glycerol release from adipose tissue fragments. Epinephrine together with resveratrol increased lipolysis by further a 28% compared to epinephrine-stimulated lipolysis and 72% above basal glycerol release [65].

Gómez-Zorita *et al.* carried out a study to analyze the acute effects of resveratrol on lipolysis and glucose uptake in mature adipocytes obtained from overweight women subcutaneous abdominal adipose tissue undergoing reconstructive surgery [66]. Adipocytes were incubated for 4 h with resveratrol (0.1 μM, 10 μM). After incubation, basal lipolysis and lipolysis stimulated with isoprenaline (10 nM), a β-adrenergic agent, were determined. Under these experimental conditions, resveratrol on its own did not promote glycerol release. However, following adrenergic stimulation, at the dose of 10 μM it stimulated glycerol release, showing only a slight tendency to do so at the lower concentration (0.1 μM).

*In vivo* studies, carried out using rats, have been performed by our group in order to determine the lipolytic effect of resveratrol. In the study by Alberdi *et al.*, resveratrol administered at a dose of 30 mg/kg/d significantly reduced epididymal adipose tissue. This led to a significant increase in HSL gene expression, without changes in ATGL mRNA levels [47]. In another study using obese Zucker rats (*fa/fa*), a model of genetic obesity, the reduction in adipose tissue mass induced by resveratrol administered at 15 mg/kg body weight/day for 6 weeks was accompanied by increased HSL mRNA levels in epididymal adipose tissue with no changes either in ATGL or SIRT-1 gene expressions [45].

Taken as a whole, the reported results demonstrate that resveratrol *per se* only shows lipolytic effect when added to the incubation medium at the highest concentrations (50–100 μM) (Figure 1). At any concentration analyzed it enhances β-adrenergic-stimulated lipolysis. To date there is no general consensus concerning the roles played by ATGL and HSL in this effect as diverse results have been obtained in *in vitro* or *in vivo* studies, or even when different strains have been used in animal studies.

## 6. Effects of Resveratrol on Thermogenesis

Heat production in response to environmental temperature or diet, is defined as adaptative or facultative thermogenesis [67]. It is mediated by uncoupling proteins (UCPs), which are located in the inner mitochondrial membrane. These proteins act as uncouplers of oxidative phosphorylation by dissipating the proton gradient across the membrane and producing heat, rather than being used to drive the synthesis of ATP [68]. UCPs can, this way, dissipate surplus caloric energy and consequently can be important regulators of body weight.

Very little has been reported in the literature concerning the effects of resveratrol on this metabolic process (Table 5). In the study carried out by our group in rats fed an obesogenic diet and treated with resveratrol at a dose of 30 mg/kg body weight/d for 6 weeks, we observed that this polyphenol induced a significant increase in protein expression of UCP1 in interscapular brown adipose tissue (IBAT), the main thermogenic tissue in rodents, and UCP3 in skeletal muscle [69]. With regard to UCP3 it is important to point out that its contribution to thermogenesis is a controversial issue. Thus, whereas several authors give support to this idea, others suggest that UCP3 is related to fatty acid oxidation [70,71].

**Table 5:** Effects of resveratrol on thermogenesis in *in vivo* studies.

Authors	Experimental Design	Effects
Alberdi *et al.* (2013) [69]	Male Sprague-Dawley rats 30 mg/kg body weight/day 6 weeks	IBAT ↑ UCP1 ↑ SIRT-1, PGC-1α and TFAM mRNA expression Skeletal muscle ↑ UCP3 ↑ TFAM mRNA expression
Lagouge *et al.* (2006) [72]	Mice	Skeletal muscle ↑ UCP3 ↑ SIRT-1, PGC-1α and TFAM mRNA expression
	400 mg/kg body weight/day	
	15 weeks	
Oliveira *et al.* (2014) [73]	Mice 1000 mg/kg body weight/day 8 weeks	IBAT ↑ UCP1 =UCP3 ↑ SIRT-1

IBAT: Interscapular brown adipose tissue; UCP: Uncoupling protein; SIRT-1: Sirtuin 1; PGC-1α: Proliferator-activated receptor-gamma coactivator 1α; TFAM: Mitochondrial transcription factor A.

In order to gain more insight into the mechanisms underlying the effect of resveratrol on UCPs, we analyzed gene expression of SIRT-1, proliferator-activated receptor-gamma coactivator-1α (PGC-1α) and mitochondrial transcription factor A (TFAM). It has been reported that resveratrol activates SIRT-1 [26], which in turn activates PGC-1α by deacetylation. This co-activator increases TFAM expression, which is a potent inducer of mitochondrial biogenesis. Moreover, PGC-1α activates transcriptional factors, such as PPARγ, retinoic acid receptor (RAR) and thyroid receptor (TR), assembled on the UCP enhancer, and thus increases UCP [67]. In IBAT resveratrol significantly increased gene expression of SIRT-1, PGC-1α and TFAM. Consequently, it can be suggested that the increased amount of UCP1 found in rats treated with resveratrol, as well as the increased mitochondriogenesis, were likely to be related to the increase in PGC-1α. In skeletal muscle a marked increase in TFAM gene expression was found, but in this case without changes in SIRT-1 and PGC-1α. However, when the ratio acetylated-PGC-1α/total PGC-1α protein was analyzed, we observed that it was decreased in resveratrol-treated rats, meaning that this co-activator was activated.

The results obtained in IBAT are in line with those previously reported by Lagouge *et al.* [72] in a study performed in mice treated with resveratrol at a dose of 400 mg/kg body weight/d for 15 weeks. The increase in skeletal muscle mitochondria and UCP3 is also in good accordance with that observed by Lagouge *et al.* [72]. However, while they found increased expression of SIRT-1 and PGC-1α in this tissue, in our study no changes were observed in these parameters. In order to understand this difference it should be pointed out that important differences between both studies exist in terms of experimental design. Thus, Lagouge *et al.* [72] used a very high dose of resveratrol (400 mg/kg body weight/day) for 15 weeks, and we used a lower dose (30 mg/kg body weight/day) for a shorter period of time (6 weeks).

Also using mice as an animal model, but with higher dose of resveratrol (1000 mg/kg body weight/day), Oliveira *et al.* reported an increase in UCP1 and SIRT-1 gene expressions in IBAT. In this study, the potential effect of resveratrol on UCP3 gene expression was also explored, but no differences were found [73].

To the best of our knowledge, only these three studies have been published investigating the effects of resveratrol on thermogenesis. According to these studies, resveratrol is able to increase UCP1 in IBAT and UCP3 in skeletal muscle (Figure 1). These effects seem to be mediated by SIRT-1. Nevertheless, information concerning this issue is scarce and further studies are needed to reinforce the scientific evidence.

## 7. Effects of Resveratrol on Fatty Acid Oxidation

An understanding of weight regulation requires a delineation of factors involved in the repartition of dietary fat between oxidation and storage. Therefore, in a body-fat reduction strategy, it is important to observe not only the balance between lipogenic and lipolytic pathways in adipose tissue, but also the lipid oxidation processes in the main oxidative tissues or organs, skeletal muscle or liver respectively [74]. In fact, a significant impairment of fatty acid oxidation might be causally involved in the development of the obese phenotype [75].

Beta-oxidation is the major process by which fatty acids are oxidized, thus providing a major source of energy. This oxidative process occurs within the mitochondrial matrix and it is catalyzed by the sequential action of four enzyme families (acyl-CoA dehydrogenase, enoyl-CoA hydratase, 3-hydroxyacyl-CoA dehydrogenase and 3-ketoacyl- CoA thiolase) [76]. Carnitine palmitoyltransferase (CPT), the key regulatory enzyme in mitochondrial β-oxidation, catalyzes the conversion of fatty acyl-CoAs into fatty acyl-carnitine molecules for entering into the mitochondrial matrix [77]. Acyl CoA oxidase (ACO) is a peroxisomal enzyme involved in fatty acid oxidation [78].

Several reports have addressed the effects of resveratrol on fatty acid oxidation either in skeletal muscle or in liver (Table 6). As far a skeletal muscle is concerned, Lagouge *et al.* in their study with C57BL/6J mice fed a high-fat diet and treated with resveratrol (400 mg/kg body weight/day) for 15 weeks, observed a decrease in total body-fat in the resveratrol-receiving animals. These animals showed larger mitochondrial structures and enhanced mitochondrial activity, which was mediated by increased gene expression of PGC1α, TFAM and UCP3 [72].

Furthermore, in a report by Alberdi *et al.* already cited in this review, we observed in Sprague–Dawley rats that resveratrol (30 mg/kg body weight/day for 6 weeks) led to increased TFAM or COX-II gene expressions in gastrocnemius muscle, suggesting increased mitochondriogenesis [79]. COX-II is a mitochondrion-encoded protein, located in the inner mitochondrial membrane, which plays a key role in the oxidative phosphorylation pathway.

**Table 6:** Effects of resveratrol on lipid oxidation in *in vivo* studies.

Authors	Experimental Design	Effects
**Skeletal Muscle**		
Alberdi *et al.* (2013) [69]	Male Sprague-Dawley rats	↑ TFAM and COX-II mRNA
	30 mg/kg body weight/day	
	6 weeks	
Lagouge *et al.* (2006) [72]	C57BL/6J mice	Larger mitochondrial structures ↑PGC-1α, TFAM and UCP3 mRNA
	400 mg/kg BW/day	
	24, 48 h	
**Liver**		
Gómez-Zorita *et al.* (2012) [56]	Male Zucker rats	↑Fatty acid oxidation
	15 mg/kg body weight/day	
	6 weeks	
Alberdi *et al.* (2013) [79]	Male Sprague-Dawley rats	↓ACO, CPT1a
	30 mg/kg body weight/day	
	6 weeks	
Ahn *et al.* (2008) [80]	C57BL/6J mice	↓Liver weight ↑PPARα gene expression
	10 mg/kg body weight/day	
	8 weeks	

TFAM: Mitochondrial transcription factor A; COX-II: Cytochrome oxidase subunit II; PGC-1α: Proliferator-activated receptor-gamma coactivator 1α; UCP: Uncoupling protein; ACO: Acyl CoA oxidase; CPT: Carnitine palmitoyl transferase PPARα: Peroxisome proliferator-activated receptor alpha.

With regard to liver, Ahn *et al.* [80] carried out a study in C57BL/6J mice fed a high-fat diet supplemented with 0.0125% of resveratrol for 8 weeks. This amount of resveratrol in the diet corresponded to a dose of 10 mg/kg body weight/day. A decrease in body weight was observed in resveratrol-treated animals. Consistent with these results, total lipids and triacylglycerols in liver were decreased. These changes were accompanied by an increase in the expression of genes related to fatty acid oxidation, such as PPARα [80].

In a study performed in our group, and previously mentioned in this review, rats fed a high-fat diet, and treated with resveratrol (30 mg/kg body weight/day) for 6 weeks showed significant decreases of CPT-1a and ACO activities in white adipose and significant increases in hepatic tissue depots [79]. Similar effects were found in another of our studies, mentioned above in this review, with genetically obese Zucker rats treated with resveratrol at doses of 15 and 45 mg/kg body weight/day for 6 weeks [56]. Thus, in both studies we suggested that increased hepatic fatty acid oxidation could contribute to the anti-obesity effect of resveratrol.

These results show that resveratrol increases fatty acid consumption in oxidative tissues (liver and skeletal muscle) by increasing mitochondriogenesis. Consequently, this polyphenol reduces adipose tissue fatty acid availability to be stored as triacylglycerols (Figure 1). The literature includes other reports showing increased mitochondriogenesis or fatty acid oxidation induced by resveratrol. These studies have not been included in the present review because they do not make any reference to changes in body fat mass.

## 8. Concluding Remarks

*In vitro* and *in vivo* studies have demonstrated that adipose tissue is the main target of resveratrol. However, lipid metabolism in liver and skeletal muscle is also affected by this polyphenol establishing a cross-talk with the adipose tissue. All the aspects of this cross-talk contribute to the anti-obesity action of resveratrol.

Summarizing the *in vitro* studies, it can be stated that resveratrol exerts its anti-obesity action by reducing adipogenesis and increasing apoptosis. With regard to adipogenesis, a wide range of concentrations have been used in the literature. There is a general consensus concerning its anti-adipogenic effect at high concentrations. In the case of low concentrations, which are close to those found in plasma and tissues from animal and humans treated with resveratrol, only a few studies have been performed and no clear conclusion can be drawn so far. Consequently, further research is needed in the form of *in vitro* studies using these physiological concentrations. As far as apoptosis is concerned, and taking into account that concentrations above 40 μM are needed to induce apoptosis, an *in vivo* effect of resveratrol on this metabolic pathway seems unlikely.

Regarding *in vivo* approaches, most of the studies have been performed in rodents. These studies show that resveratrol induces a reduction in body fat by inhibiting the fat accumulation processes and stimulating the lipolytic and the oxidative pathways. Nevertheless, extrapolation of these results to humans is a matter of concern; therefore, the effects could not be as clear as in animal models. Consequently, and taking into account that the available human studies are scarce, more studies in human beings are needed to confirm the anti-obesity effects of this polyphenol.
